# Diagnostic Challenges of Anaplastic Large Cell Lymphoma in a Resource-Limited Setting: A Case Report and Literature Review

**DOI:** 10.1155/2021/6677638

**Published:** 2021-02-11

**Authors:** Oluomachi Charity Nnachi, Innocent Paul Ezenwenyi, Augustine Ejike Okoye, Chinedu Obasi Akpa, Chukwuma Joseph Uzoigwe, Gabriel Chima Ugwu

**Affiliations:** ^1^Department of Haematology, Alex Ekwueme Federal University Teaching Hospital, Abakaliki, Ebonyi, Nigeria; ^2^Department of Histopathology, Alex Ekwueme Federal University Teaching Hospital, Abakaliki, Ebonyi, Nigeria

## Abstract

Anaplastic large cell lymphoma (ALCL) is a rare variety of non-Hodgkin's lymphoma with diverse morphologic variants. Due to the similarity of the different variants with other lymphoma entities, misdiagnosis may be inevitable when immunohistochemical and cytogenetic techniques are not available and histology alone is employed. We report a case of a 43-year-old woman with a seven-month history of neck swelling which was complicated by ulceration of the mass and pathological fracture of the right clavicle after two months delay in arriving at a correct diagnosis. Several attempts to arrive at definitive diagnosis using histology alone gave divergent reports which all misdiagnosed the case until it was sent to a facility outside the country. Our report highlights the limitations and challenges of histology in making a definitive diagnosis of ALCL and the overt importance of immunohistochemical and cytogenetic techniques which are largely unavailable in resource-constrained environment typical of tertiary centers in Nigeria and most sub-Saharan Africa countries.

## 1. Introduction

Anaplastic large cell lymphoma (ALCL) is a unique variety of non-Hodgkin's lymphoma. They are defined as a CD30-positive neoplasm of T-cell or null cell lineage and present clinically in two forms as a primary cutaneous or systemic disease with involvement of the skin, lungs, liver, soft tissues, and rarely bone [1]. ALCL has a broad morphologic spectrum with five or more variants of the disease [2, 3]. Diagnosis of ALCL is often a dilemma, and misdiagnosis may be inevitable when histology with haematoxylin and eosin alone is employed [4]. The incorporation of immunohistology has made diagnosis straightforward, but unfortunately, such techniques are still largely unavailable in our environment. We present a case highlighting the challenges encountered in the diagnosis of ALCL using histology alone.

## 2. Case Report

**Figure 1:**
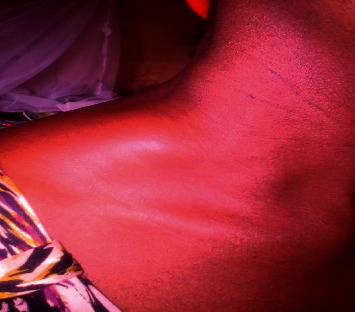
Photograph of neck mass at the first presentation.

**Figure 2:**
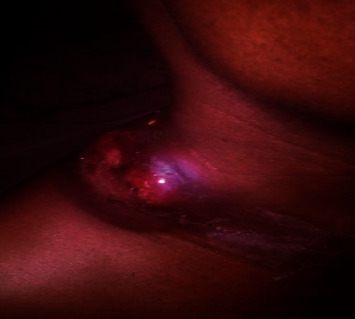
Ulceration of neck mass.

**Figure 3:**
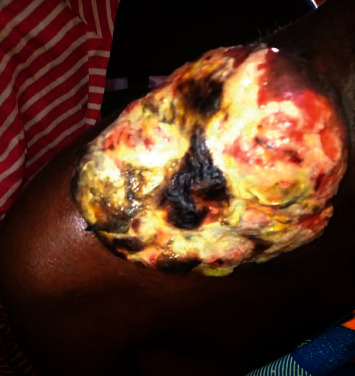
Extensive ulceration of neck mass while a diagnosis is awaited.

**Figure 4:**
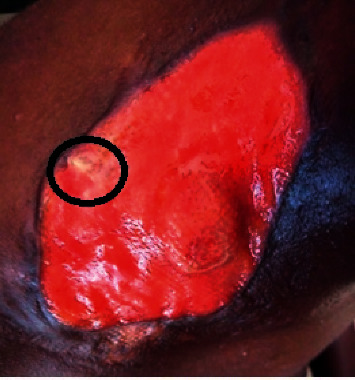
Protrusion of clavicular fracture end from the ulcer surface.

A 42-year-old female Nigerian civil servant presented to the adult haematology unit of Alex Ekwueme Federal University Teaching Hospital, a tertiary health facility in Ebonyi State, South-East Nigeria, in January 2019 with a seven-month history of painless right-sided neck swelling (Figure 1), fever, general body weakness, drenching night sweats, and weight loss. According to medical history, she had sought care at the internal medicine unit of our facility where she received prescription of several antibiotics, antiviral, and antituberculosis medication following a biopsy of the mass and histology report of reactive lymphadenopathy. After completing her medications, the neck swelling regressed partially. Four months later, she returned to her physician with complaints of weakness, resurgence, and rapid progression of the neck mass. On further interrogation, she was noted to be anaemic, hence necessitating referral to the haematology clinic. On presentation at our clinic, clinical examination revealed a neck mass measuring 8 × 6 cm (Figure 1) at the right supraclavicular region. The patient reported no pain related to the mass. There was generalized lymphadenopathy involving cervical, supraclavicular, and axillary lymph nodes (largest measuring 5 × 3 cm). Two months after presentation at the haematology clinic, the right supraclavicular mass ulcerated with exudation of straw-colored fluid and development of a pathological fracture of the right clavicle (Figures 2–4).

Laboratory tests revealed a complete blood count with haemoglobin of 5 g/dl, with normal white blood cell and platelet parameters. ESR was 95 mm/1st hour. Peripheral blood film showed few small- and medium-sized matured lymphocytes with nuclei cleave. HIV, hepatitis B surface antigen, and hepatitis C tests were negative. An acid-fast bacilli test for the fluid from the ulcerated mass and Mantoux test were negative, while chest X-ray showed no abnormality. Renal and liver function tests had normal values. Neck ultrasound scan revealed cervical, supra-, and infraclavicular lymph node enlargements. Abdominal ultrasound showed hepatomegaly (17.2 cm), intra-, and retroperitoneal lymph node enlargement (largest measuring 4.2 cm by 1.1 cm).

The previous biopsy block of the neck mass was retrieved, and a repeat biopsy was also done and both were sent for another histology opinion. This second request made a diagnosis of small lymphocytic lymphoma (SLL) with microscopy showing sections of diffused sheets of matured lymphocytes growing in solid sheets and effacing the lymph node architecture without necrosis. Immunohistochemistry could not be done because it was not available.

**Figure 5:**
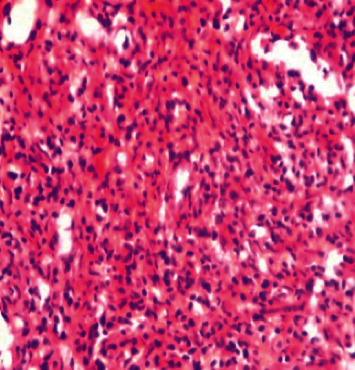
Third histology report of Hodgkin's lymphoma (haematoxylin and eosin stain).

The diagnosis of SLL was refuted by the managing haematologists. They argued that the patient's clinical course, especially the clavicle fracture, was uncommon in SLL. Due to this dilemma, a third histology opinion was requested from another referral pathology facility. This report noted the presence of numerous mononuclear and classic variants of Reed–Sternberg with prominent inflammatory cell components particularly eosinophils and neutrophils. Hence, a diagnosis of Hodgkin's lymphoma, mixed cellularity variant, was made (Figure 5).

Occasioned by divergent histology diagnoses from three different referral facilities, unavailability of immunohistochemical techniques, and rapid deterioration of patient's clinical condition, the specimen had to be shipped to another facility outside the country for repeat histology and immunohistochemistry. After a period of four weeks, the report of the fourth histology request was received. The microscopic findings revealed significant plasmacytic infiltrate with some of the cells having a resemblance to Reed–Sternberg cells and many others that have nuclei with embryonic shape. Immunohistochemical staining of the Reed–Sternberg-like and cells with embryonic shaped nuclei showed positivity for CD 30 and ALK but are negative for EMA, CD 45, EBV-LMP 1, PAX 5, CD15, and CD 3. This report led to the diagnosis of anaplastic large cell lymphoma.

**Figure 6:**
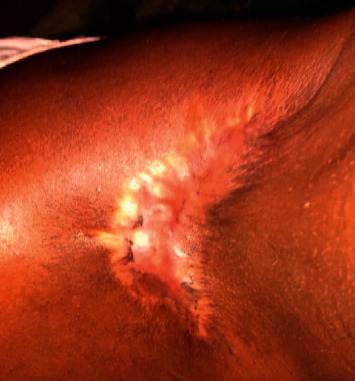
Postchemotherapy and radiotherapy aspect showing total regression of neck mass, fracture, and wound healing.

The patient was counseled and prescription ordered for chemotherapy. She received 9 cycles of cyclophosphamide, doxorubicin, vincristine, and prednisolone, wound dressing, physiotherapy, and involved-field radiotherapy. Blood parameters were monitored throughout the treatment period. During chemotherapy and radiotherapy, tumour regression was observed and total ulcer healing achieved after the ninth course. She showed complete response with a restoration of normal haemoglobin, ESR values ranging between 3 and 8 mm/1st hour, clavicle fracture and wound healing (Figure 6). Right upper limb movement has also been restored, and the patient is currently in remission (twelve months at the time of this report).

## 3. Discussion

Anaplastic large cell lymphoma (ALCL) is a malignancy of mature T lymphocytes with a morphologic expression of large atypical lymphoid cells with abundant cytoplasm, pleomorphic horseshoe-shaped nuclei, and prominent nucleoli [5]. This lymphoma carries malignant clones characterized by membrane CD30 positivity as a constant immunophenotypic signature and has a duality of clinical manifestation, namely, primary cutaneous ALCL with a sole skin manifestation and systemic ALCL with a multiorgan manifestation involving soft tissues and lungs, while bone involvement is rare [6]. The presence or absence of anaplastic lymphoma kinase (ALK) expression further classifies the ALCL into ALK-positive or ALK-negative which has prognostic significance. ALK-positive and ALK-negative lymphoma are morphologically similar but distinguishable on immunostaining. ALK-positive cases have translocation involving the ALK gene and expression of ALK protein. Cutaneous ALCL is mostly T-cell-derived but differs from systemic ALCL because it is always ALK-negative [7, 8].

Systemic ALK-positive ALCL is a disease with complex morphological heterogeneity that stems from its eclectic tumour cell cytology. The histologic variants include the common, lymphohistiocytic, small cell, Hodgkin-like, and composite patterns [7, 8].

Typically, ALCL evolves histologically in a manner that brings about incomplete effacement of the lymph node architecture with a proclivity of the tumour cells to make incursions into the lymph node sinuses mimicking a metastatic pattern. In the background, there is a potpourri of inflammatory cells comprising plasma cells, histiocytes, and eosinophils in the company of the malignant clones. Sometimes, the inflammatory cells are preponderant and may obscure the tumour cell population making diagnosis even more cumbersome.

The common type consists of sheets of large pleomorphic tumour cells with horseshoe-shaped or kidney-shaped nuclei, while the small cell type is characterized by small- and medium-sized pleomorphic tumour cells. The lymphohistiocytic variant consists of both small and large tumour cells forming rosettes around blood vessels in a background replete with reactive histiocytes. This background creates a diagnostic hurdle with a tendency for misdiagnosis of this subtype as atypical inflammatory lesion or haemophagocytic syndrome. The Hodgkin-like ALCL is morphologically characterized by the presence of Reed–Sternberg-like cells and shows lymph node capsular thickening and nodular fibrosis.

These morphological features that typify the different forms of ALCL were highlighted in one report or the other of the histological diagnoses made in the index case by the anatomical pathologists. Despite that, the precise disease entity was not identified due to the limitations posed by the use of haematoxylin and eosin as the sole diagnostic technique.

The clinical course of disease in the index case was not in tandem with the various histological diagnoses made, and this was further complicated by the bone involvement which is uncommon in non-Hodgkin's lymphoma. The danger of the employment of morphology alone in diagnosis lies in its inability to highlight the nuances inherent in the various lymphoma types with morphologic similarity, and further techniques are requisite for diagnostic precision.

Immunohistochemistry is invaluable in the differential diagnosis of lymphoma entities with cytomorphologic similarity to ALCL [9, 10].

Currently, diagnosis, classification, and proper treatment of lymphoma deeply depend on the detection of cellular markers and the use of genetic analysis of tissue samples to support histologic and clinical findings. These diagnostic modalities include immunohistochemistry (IHC) and/or flow cytometry, cytogenetic, and molecular analyses [8]. These techniques have become the crux of diagnostic management of lymphoma in recent times.

ALCL, though mostly T-cell-derived, displays T, B, and null immunophenotypes. Immunohistochemical staining for the following markers: ALK, CD30, CD5, CD3, CD7, CD2, CD4, CD15, CD20, EMA, and cytotoxic markers (TIA1, granzyme B, PAX5, PGMI, EBV, and perforin) is vital in the differential diagnosis of ALCL.

Consistent immunophenotypic features of most cases of ALCL include positivity to CD30, ALK, granzyme B, TIA-1, and perforin and negativity to CD20, EMA (positive in diffuse large B-cell lymphoma), CDI5 (positive in Hodgkin's lymphoma), and CD3 (positive in peripheral T-cell lymphoma).

Cytogenetic and molecular analyses in ALCL detect molecular defects which may be translocations, gene rearrangements, or recurrent genetic abnormalities. ALK protein is a normal protein coded for by the ALK gene located in chromosome 2. However, with a chromosomal translocation between chromosome 2 and chromosome 5 (t (2 : 5) (p23 : q35)), the ALK gene is brought in proximity with the nucleophosmin (NPM) gene. The NPM-ALK fusion gene formed confers constitutive activity on the tyrosine kinase receptor.

TP63 rearrangement along with the coexpression of CRBB4 and COL29AL is another molecular abnormality that is noted in ALCL. Experimental data suggest that targeted drug therapy against the coexpressed factors inhibits tumour growth and slows disease progression.

Moreover, recurrent chromosomal aneuploidies with additional 17q, 5q, 6p, 8q, 12q, Iq or loss of 4q, 1 Iq, or 13q and defects of the JAK-STAT pathway have also been implicated in the pathogenesis of ALCL.

This case report exposes the inadequacy of H&E alone in the diagnosis of lymphoma cases, but unfortunately, it is the readily available technique in our environment. More advanced techniques such as IHC, fluorescence in-situ hybridization (FISH), and reverse transcriptase-polymerase chain reaction (RT-PCR) are not common in many tertiary or referral health institutions in Nigeria and sub-Saharan Africa at large [11, 12]. This has brought about a humongous limitation to accurate diagnosis and classification of diseases and has made treatment prone to error.

The 2011 report of the International Network for Cancer Treatment and Research on the evaluation of infrastructure for lymphoma corroborated the fact that IHC, cytogenetics, and FISH techniques are unavailable in most parts of Nigeria and other sub-Saharan African countries [13].

Incidence of non-Hodgkin's lymphoma in sub-Saharan Africa is reported to be as low as 30,000 and as high as 278,000 cases each year [14, 15]. The growing burden of lymphoma fostered by population growth and human immunodeficiency virus infection is projected to be detrimental on the continent with grossly limited current diagnostic services. The burden of lymphoma may be detrimental on the continent with grossly limited current diagnostic services. In order to circumvent the diagnostic challenges posed by infrastructural inadequacy, most sub-Saharan African nations resort to sending tissue samples abroad for holistic analysis and accurate diagnosis. This approach is marred by extra cost, tissue loss in transit, increased turn-around time, delay in treatment, disease progression, loss of learning opportunity, and diminished academic interaction.

## 4. Conclusion

The diagnostic quagmire posed by the broad morphologic spectrum of ALCL is magnified in the setting of inadequate diagnostic techniques as found in our setting and also in most tertiary treatment centers in sub-Saharan African countries. Efforts should be geared towards the provision of these techniques, manpower training, and collaboration to strengthen our treatment centers to be better equipped to diagnose, classify, and institute appropriate timely treatment for lymphoma.

## Data Availability

The data used for the findings of this article are available and will be provided by the corresponding author upon request.
